# Cadmium Increases the Sensitivity of Adolescent Female Mice to Nicotine-Related Behavioral Deficits

**DOI:** 10.1155/2014/360978

**Published:** 2014-11-16

**Authors:** Philip Adeyemi Adeniyi, Babawale Peter Olatunji, Azeez Olakunle Ishola, Duyilemi Chris Ajonijebu, Olalekan Michael Ogundele

**Affiliations:** ^1^Department of Anatomy, Cell Biology and Neuroscience Unit, College of Medicine and Health Sciences, College Building II, Afe Babalola University, Room G14, KM 8.5 Afe Babalola Way, PMB 5454, Ado-Ekiti, Ekiti State, Nigeria; ^2^Department of Biological Sciences, College of Sciences, Afe Babalola University, Ado Ekiti, Nigeria; ^3^Department of Anatomy, College of Health Sciences, University of Ilorin, Ilorin, Nigeria; ^4^Department of Physiology, College of Medicine and Health Sciences, Afe Babalola University, Ado Ekiti, Nigeria

## Abstract

This study investigates spatial and nonspatial working memory, anxiety related behavior, and motor activities in cadmium and/or nicotine exposed female adolescent mice. P28 female adolescent mice (albino strain) were divided into four groups of five (*n* = 5) mice each. A set of mice (Nic) received subcutaneous nicotine (2.0 mg/kg) while a separate set (Cd) was treated with 2.0 mg/kg cadmium (subcutaneous). For the combined treatments of cadmium and nicotine, we administered 2.0 mg/kg Nicotine and 2.0 mg/kg of Cd. Subsequently, a separate group of animals (*n* = 5; control) received normal saline. The total duration of treatment for all groups was 28 days (P28–P56). At P56, the treatment was discontinued, after which the animals were examined in behavioural tests. Nicotine and cadmium increased the metabolism and food intake in the female adolescent mice. This also corresponded to an increase in weight when compared with the control. However, a combined nicotine-cadmium treatment induced a decline in weight of the animals versus the control. Also, nicotine administration increased the motor function, while cadmium and nicotine-cadmium treatment caused a decline in motor activity. Both nicotine and cadmium induced a reduction in memory index; however, nicotine-cadmium treatment induced the most significant decrease in nonspatial working memory.

## 1. Introduction

Nicotine, a cholinomimetic alkaloid, plays an important role in the neurophysiologic changes which lead to dependence [1, 2]. Nicotine is the main active ingredient in tobacco smoke that causes and maintains tobacco addiction [3]. Many studies have revealed that nicotine produces tolerance and leads to psychological and physical dependences in adults [4]. Nicotine has been reported to increase 5-hydroxytryptamine (serotonin, 5-HT) release in the cortex, striatum, hippocampus, dorsal raphe nucleus (DRN), hypothalamus, and spinal cord [5–7]. Also nicotine induces the release of several other neurotransmitters, including acetylcholine (ACh), noradrenaline, GABA, and glutamate [4, 8–11]. Although the mechanism of nicotine-related tobacco smoke addiction is unclear, it is suspected to involve several chemical pathways, neurotransmitter, and anatomical changes in different brain areas [4, 12, 13].

The most predominant method of nicotine use is the inhalation of tobacco smoke which consists of nicotine, cadmium, and other alkaloids [14, 15]. Interestingly, studies have shown that the effect of nicotine administered alone, on behavior, differs from the observed behavioral changes when it was coadministered with cadmium in tobacco smoke [16, 17]. On its own, cadmium is a long-standing heavy metal present in the tobacco leaf and the environment, with an average concentration of 0.1 and 0.5 parts per million (ppm) [18]. Following prolonged exposure, coupled with its ubiquitous nature, bioaccumulation and toxicity of cadmium increased in the body of smokers [18, 19]. In addition, exposure through inhalation or ingestion of agroproducts (phosphate fertilizers) often amounts to the accumulation of cadmium up to a concentration of 300 mg/kg [18, 20, 21].

In smokers, serum level of cadmium and nicotine is usually higher than that observed in nonsmokers [22, 23]. With both having differential signaling pathways, neurotoxicity is a common action of nicotine, cadmium, and their metabolites. Although nicotine and nicotinic receptors are naturally existing modulators in the central nervous system, use of nicotine from tobacco smoke often increases the concentration of nicotine in the blood and brain tissue causing the observed toxicity [24, 25]. In effect, several neurotransmitters (dopamine, 5HT, acetylcholine, GABA, glutamate, and epinephrine) are upregulated [26, 27], while signaling molecules like prolactin are suspected to be deregulated [28]. Consequently, this translates into behavioral changes and addiction in the animals. As a result of inhalation of nicotine in tobacco smoke, the presence of cadmium in the smoke plays an important role in the behavioral changes observed in prolonged use and addiction [27, 29]. In addition, studies have shown that anatomical modifications and degenerative changes occur in specific brain regions after prolonged tobacco smoke addiction [30]. This has been attributed to the persistent excitotoxicity of cholinergic receptors through nicotine potentiation and cadmium-mediated reactive oxygen species (ROS) production (cadmium inhibits cytochrome a3 and cytochrome C oxidase) [29, 30].

It is evident, from previous findings, that 60% of smokers start before the age of 14 (adolescent) and 90% show dependence before the onset of adult hood [31]. In addition, females have shown more sensitivity to nicotine use than males and are described as being more vulnerable to nicotine reward than males [31, 32]. Despite the striking evidence of the involvement of tobacco smoke on behavior and addiction in adolescent female, the exact interaction between nicotine and cadmium in addiction and toxicity is yet to be elucidated. Using behavioral tests, we have studied the effect of nicotine and/or cadmium on weight, motor activity, and memory function in female adolescent mice to demonstrate the role of cadmium in the observed nicotine susceptibility and weight loss seen in adolescent female mice.

## 2. Materials and Methods

### 2.1. Drugs

Nicotine bitartrate was generously given by Dr. Maria A. Deluca of Department of Biological Science, University of Cagliari, Cagliari, Italy, and cadmium chloride was obtained from Department of Chemical Sciences, Afe Babalola University, Nigeria. The salts were dissolved in saline (Drugfield Pharmaceutical LTD, Nigeria) and doses are expressed as free base.

### 2.2. Animal Preparations and Treatment

*N* = 20 female adolescent mice (P28) were procured form the animals holding facility of Afe Babalola University. The animals were kept under standard laboratory conditions of 12-hour alternating light and dark, controlled temperature, and adequate ventilation. They were fed on standard rat chow containing proteins, carbohydrate, fats, vitamins, minerals, and water ad libitum. The animals weigh between 9 and 12 gm and were separated randomly into four (4) groups of *n* = 5 animals each. Nicotine (Nc) was administered subcutaneously (2 mg/Kg) to a set of *n* = 5 animals, while a separate group received cadmium (Cd; Subcutaneous) at a dose of 2 mg/Kg [33, 34]. A combined treatment group was set up and was treated with cadmium (2 mg/Kg) and nicotine (2 mg/Kg) (Nc + Cd), while the control (vehicle) received normal saline for the period of the treatment (28 days; chronic administration). The nicotine and/or cadmium were administered between 9 and 10 am on each day of the treatment from P28 to P56. All protocols were approved by Afe Babalola University Animal Care Committee following the IACUC guidelines for animals use in research and education.

### 2.3. Weight Measurements

The weight of the animals was measured daily for the duration of the experiment. Also, the daily food intake was measured using metabolic specially designed metabolic cages.

### 2.4. Behavioral Assays

The behavioral tests were carried out in a closed area with proper illumination and sound control. All behavioral test performance was captured using a digital video recorder and analyzed in Any-Maze software. The animals were trained in the testing area 3 days to the actual test and were allowed to acclimatize to the equipment used for the various tests.

### 2.5. Rotarod Test

The animals were placed on a rotarod to determine the latency of fall for three separate trials (T_1_, T_2_, and T_3_) during which the speed of the rotarod was gradually increased from 4 rpm to 35 rpm. The maximum duration allowed for each trial was 3 minutes.

### 2.6. Novel Object Recognition Test (NOR)

This was done to evaluate the nonspatial working memory of the animals. For the first trial (T_1_) animals were placed in a white opaque test area with two identical objects for 5 minutes following which an intertrial time (IT) of 60 minutes was observed. A second trial (T_2_) was administered during which one of the old objects was replaced with a novel object for 5 minutes in the same test area. The performance of the animals was recorded and analyzed to determine the exploration time on old and new objects in T_2_, respectively. Subsequent analysis involves the plotting of the memory index using the method of Kruk-Słomka et al. [35].

### 2.7. Y-Maze

This was done to check the spatial working memory of the animals. The rats were placed facing the edge and allowed making their arm decision for total test duration of 4 minutes. The frequencies of alteration between the arms were recorded as plotted to determine the memory index (percentage alteration).

### 2.8. Elevated Plus Maze (EPM)

Using an elevated plus maze, anxiety linked behaviors were assessed in the animals (*n* = 5 per group). These include the frequency of head dipping (HD), open arm duration (OAD), closed arm duration (CAD), closed arm entries (CAE), and open arm entries (OAE). The duration of the test was 4 minutes per mice.

## 3. Statistical Analyses

The recorded video performance was analyzed using Any-Maze software to determine object exploration time (NOR), percentage arm alteration (Y-maze), and latency of fall (rotarod). Open and closed arm parameters were measured for elevated plus maze. The data was plotted in one-way ANOVA (GraphPad Prism Version 6.0) with Bonferroni post-hoc test. Significance was set at ^*^ *P* < 0.05 and results were expressed as means ± standard error of the mean (SEM). Spearman's correlation was used to analyze the average weight gained per day and average food intake per day from P28 to P56.

## 4. Results

### 4.1. Weight Changes

**Figure 1:**
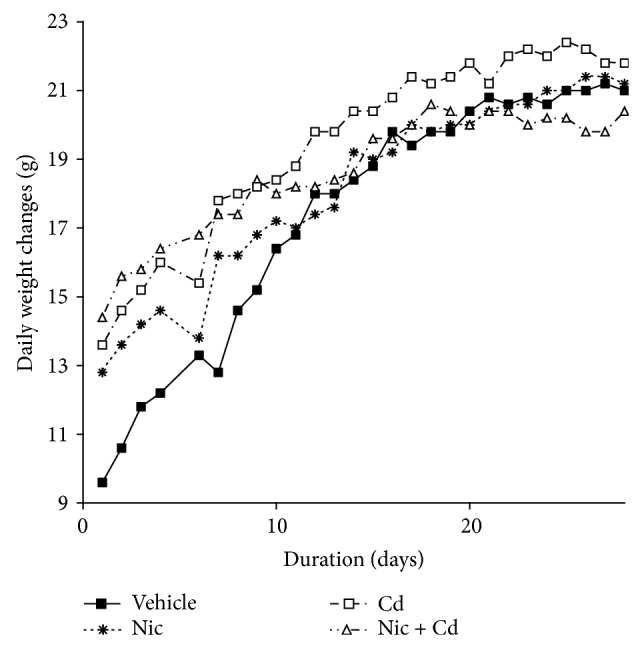
Spearman's correlation showing the time-dependent weight changes in the treatment groups and control. The nicotine and/or cadmium treatment caused a significant increase in average weight from P28 to P56 when compared with the control. It was observed that nicotine-cadmium treatment induced a decline in weight around P37 versus saline administered mice statistically. This is suggestive that the weight loss associated with tobacco use is not an exclusive effect of nicotine, but an effect of combined nicotine and cadmium in tobacco smoke.

In order to evaluate the effect of nicotine, cadmium, or a combined treatment on the development of the adolescent female rats, we measured the weight, daily, for the duration of the experiment. Consequently, we observed a significant increase in weight of the animals in the cadmium, nicotine, and Nc + Cd group when compared with the control (*P* < 0.05). Progression correlation shows that the cadmium and nicotine treated groups recorded the highest increase in weight from P28 to P56 while the combined treatment with nicotine-cadmium recorded a decline from P46 to P56 when compared with the control (Figure 1).

### 4.2. Daily Food Intake

**Figure 2:**
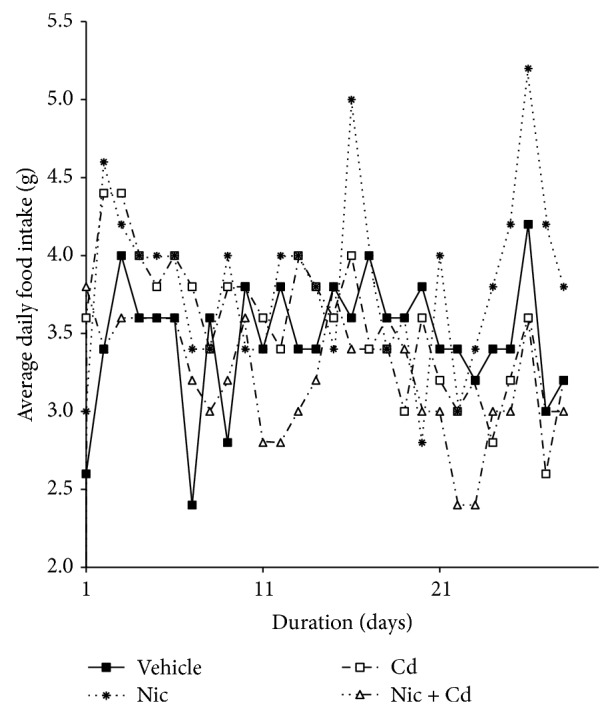
Average daily food intake (P28 to P56) in female adolescent mice. Similar to the observations in weight changes, the average daily food intake increased in the nicotine and cadmium treated groups when compared with the control. Consequent of nicotine-cadmium treatment, a decline in food intake was observed around P46 versus the saline treated mice. This precedes the weight loss in this group (P50) as shown in Figure 1. Each point represents daily recorded food intake/group expressed as mean ± SEM.

To elucidate the effect of nicotine and/or cadmium on metabolism (calorie intake) during the adolescent stage of female development, the daily food intake of each group was recorded for the duration of the experiment. Similar to our observations on weight changes, the nicotine treated animals recorded the most significant increase in food intake rate when compared with the control (*P* < 0.01^∗∗^). Interestingly, the cadmium treated animals recorded an increase in food intake (*P* < 0.05^∗^) but was less significant versus the nicotine treated group. In addition, cotreatment with nicotine-cadmium recorded a steady decline in food intake around P30–P56. This also correlated with the decline in weight observed in this group versus the control around P46 (Figure 2).

### 4.3. Memory Function

#### 4.3.1. Y-Maze

**Figure 3:**
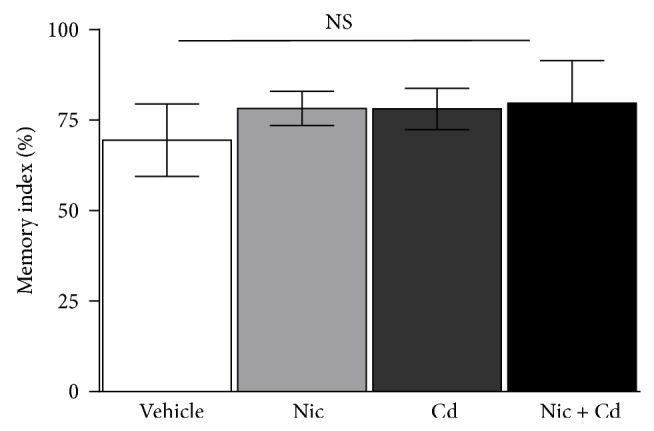
Percentage arm alteration expressed as memory index in Y-maze. No statistical significance was observed in spatial memory function for the treatment groups when compared with the control. Empirically, slight increase was seen in all treatments with the highest recorded in the combined nicotine-cadmium treatment.

This was used to analyze the spatial working memory in the female adolescent mice after an exposure to nicotine, cadmium, or a combination of both (nicotine-cadmium) during the adolescence period (P28–P56). We evaluated the percentage alteration between the arms A, B, and C and plotted the memory index (MI). No significant change in spatial memory function was observed when the treated groups were compared with the control (Figure 3).

#### 4.3.2. NOR

**Figure 4:**
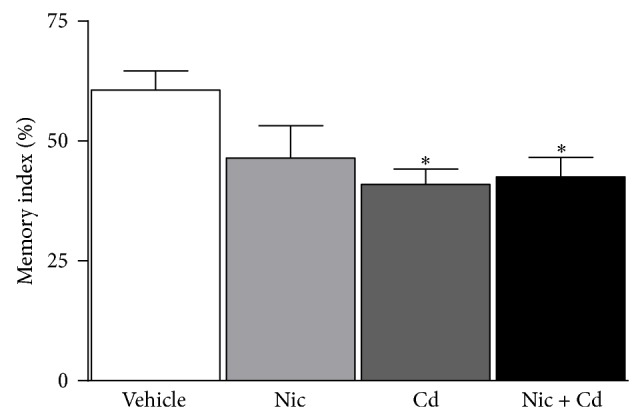
Novel object recognition memory index. A significant decline in memory index was observed in the cadmium and combined (nicotine-cadmium) treatment groups (*P* < 0.05) when compared with the control. Although nicotine treatment recorded no statistically significant decline in memory index, empirical quantification shows a decline in the memory index versus the control. However, comparing the nicotine treated group with the combined treatment group demonstrates the effect of cadmium in facilitating decline in memory function when coadministered with nicotine, similar to what is observed in tobacco smoke (*statistical significance*: ^*^ *P* < 0.05).

The exploration time on old and new objects was determined and used in calculating the memory index. Although no statistically significant decline in memory index was observed in the nicotine treated group, empirical quantification shows a reduction in memory index versus the control. Furthermore, the cadmium treatment and the combined treatment group showed a decline in memory function (*P* < 0.01) (Figure 4).

#### 4.3.3. Rotarod

**Figure 5:**
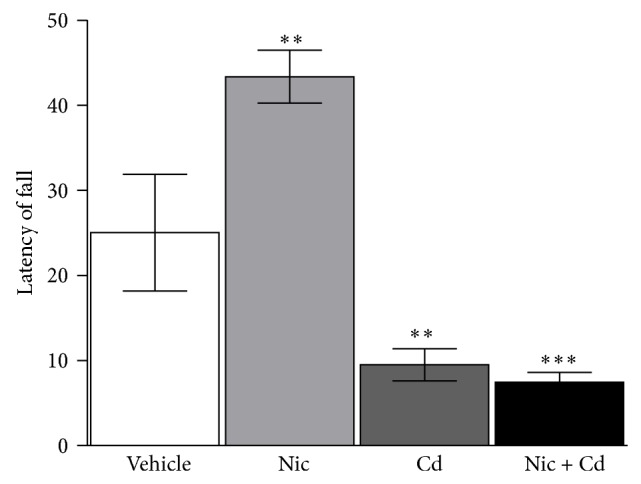
Latency of fall (LOF) in rotarod test for motor function. Treatment with nicotine increased the latency of fall significantly (↑motor function) when compared with the control group (*P* < 0.05). This is attributed to the role of nicotine in stimulating cholinergic receptors at neuromuscular junctions and increasing epinephrine release. The cadmium (*P* < 0.01) and nicotine-cadmium (*P* < 0.001) treated groups showed a significant decrease in motor function (↓LOF) when compared with the control and nicotine treated group (*P* < 0.001). Subsequent analysis shows that nicotine-cadmium treatment reduced motor function significantly when compared with nicotine (*P* < 0.001) and cadmium (*P* < 0.05) treatment groups, respectively (*statistical significance*: ^*^ *P* < 0.05, ^**^ *P* < 0.01, and ^***^ *P* < 0.001).

The latency of fall (LOF) was evaluated for *n* = 5 animals in each group. Three (3) trails T_1_, T_2_, and T_3_ were conducted for each animal for duration of 3 minutes per trial. An intertrial time (IT) of 12 minutes was also observed between the consecutive tests. The average time spent on the rotarod for the three trials was determined as the representative value for each animal. A significant increase in LOF was observed in the nicotine treated group (*P* < 0.05) when compared with the control. However, cadmium and nicotine-cadmium treatment caused a significant decrease in motor function, recording a decrease in LOF when compared with the control (*P* < 0.01) and the nicotine treatment group (*P* < 0.001). Similar to our findings in memory function, a combination of nicotine and cadmium induced a decline in motor function significantly when compared with nicotine (*P* < 0.001) and cadmium (*P* < 0.05) (Figure 5).

#### 4.3.4. Elevated Plus Maze (EPM)

**Figure 6:**
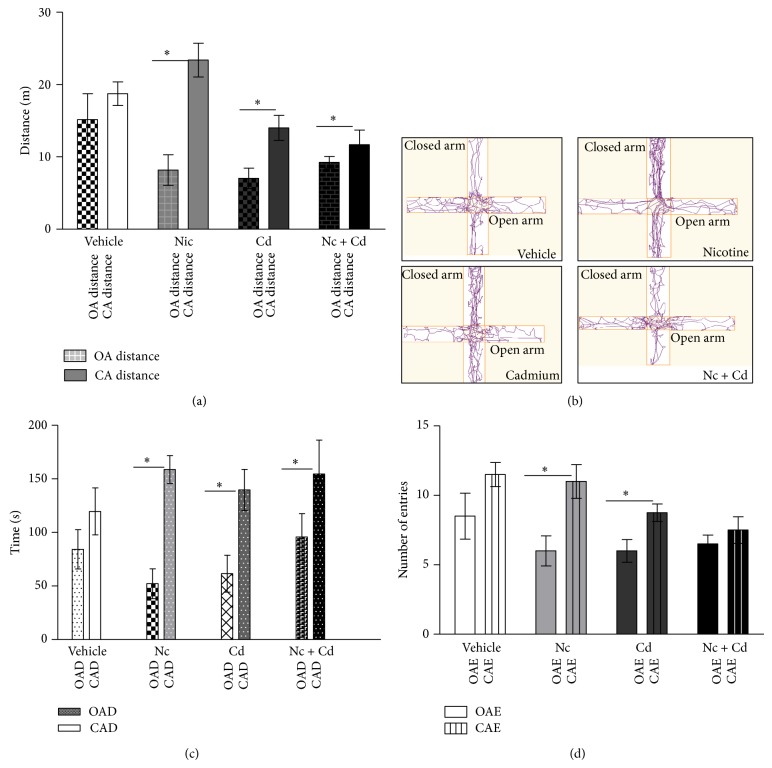
(a, b) The average distance travelled in meters in elevated plus maze. (a) The open arm distance (checked bars) and closed arm distance (plain bars) for the treatment groups were compared versus the control. Nicotine treated female mice showed a significance in the distance covered in the open arm when compared with the control (*P* < 0.05). In furtherance, cadmium treated mice recorded a decrease in total exploration distance when compared with the nicotine treated group (*P* < 0.05). Anxiety linked behaviors were observed in the nicotine treatment as they explored the closed arm more than the open arm. Also, the cadmium and nicotine-cadmium treated groups showed a significant decline in exploration time and explored the closed arm more than the open arm (*statistical significance*: ^*^ *P* < 0.05). (b) Track plots of the mice during the 4 minutes exploration in EPM using Any-Maze software to demonstrate the open and closed arm distances. (c and d) Elevated plus maze open/closed arm entries and duration. (c) Close arm (CAD::stripes) and open arm (OAD::plain) durations for the treatment groups versus the control. Increased CAD was observed in the nicotine, cadmium, and nicotine-cadmium treatment group (*P* < 0.05), thus depicting increased anxiety in these animals. (d) Bar chart showing the frequency of close arm (CAE) and open arm (OAE) entries for the treatment groups and control. A significant difference (*P* < 0.05) was observed between the CAE and OAE in the nicotine and cadmium treated groups (^*^ *P* < 0.05). No significance was seen when the OAE and CAE were compared for the combined nicotine-cadmium treatment group (*statistical significance*: ^*^ *P* < 0.05).

We employed the behavioral assay technique to detect anxiety related behaviors in the female adolescent mice after nicotine and/or cadmium treatment. There was a statistical significance in the open and closed arm distance for the treatment groups when compared with the control (*P* < 0.05) (↑anxiety). The nicotine (Nc) and cadmium (Cd) treated groups recorded a significant increase in closed arm distance (↑anxiety) versus the open arm distance for the same group and the control (OA and CA distance) (Figures 6(a) and 6(b)). The combined treatment (nicotine-cadmium) showed a reduction in exploration distance for both open and closed arms (Figure 6(b)); however, it also recorded a more significant closed arm distance versus the open arm distance (↑↑anxiety; *P* < 0.01). We next analyzed the duration spent by each group in either the open or closed arms to further determine the magnitude of anxiety. Similar to our findings in the arm distance analysis, the treatment groups recorded significantly higher durations in the closed arm versus the open arm (↑anxiety) when compared with the control (*P* < 0.05). Similarly, the most significant increase in CAD was observed in the nicotine-cadmium treatment (↑↑anxiety) (Figure 6(c)). Analysis of the frequency of open and closed arm entries further supports these findings as the combined treatment (nicotine-cadmium) showed more anxiety with the least rate of total arm entries (decreased exploration) (Figure 6(d)).

## 5. Discussion

Taken together our findings suggest that nicotine or cadmium treatment induced an increase in food consumption rate in the female adolescent mice. In addition, a steady increase in weight was observed in these groups when compared with the control. However, the nicotine-cadmium treatment showed a decline in food consumption rate and weight from P37 to P56, suggesting the negative effect of this combination on metabolism and development. Furthermore we observed that nicotine increased motor function in the mice when compared with the control while cadmium induced a decline in motor function. However, coadministration of nicotine-cadmium induced the most significant decline in motor function versus the control. We have also shown that nicotine administered between P28 and P56 caused no significant decline in memory function for spatial (Y-maze) and nonspatial (NOR) memory while cadmium induced a decline in memory function. Similar to our observations in motor function, a combination of nicotine-cadmium treatment induced the most significant decline in memory function; especially in novel object recognition memory. Taken together we deduce that nicotine, when singly administered, holds the potential of reducing memory function after prolonged use. However, its combination with cadmium induces a significant decline in memory function over a short period of time. Similarly, cadmium administration induced a more significant decline in nonspatial working memory when compared with the nicotine-cadmium and control.

Anxiety linked behaviors were also observed both in the nicotine and treatment groups (Figure 6(b)). In EPM, the nicotine and cadmium treatment groups explored the closed arm of the maze more than the open arm (*P* < 0.05) when compared with the control. However a combined treatment of nicotine and cadmium caused a decrease in exploration distance in the closed arm of the maze. This also corresponded to an increase in closed arm duration, thus depicting anxiety in this treatment group (reduced exploration distance and prolonged closed arm duration) (Figures 6(a)–6(c)). The findings from EPM show that both nicotine and cadmium can induce anxiety-linked behaviors after prolonged use, a combined treatment of nicotine-cadmium, similar to the tobacco smoke, caused a significant increase in anxiety in the female adolescent mice. Other anxiety linked symptoms observed in the combined treatment group (nicotine-cadmium) include an increased food consumption, decreased weight, and a decline in working memory.

Studies have shown that nicotine, a cholinergic agonist, is capable of altering the rate of food consumption and energy expenditure, thus leading to weight changes [36, 37]. Although the exact mechanism remains elusive, both central and peripheral mechanisms are suspected to be involved. The significant effect of nicotine on metabolism has been described to occur through its ability to potentiate several cholinergic receptors in the central nervous system (hypothalamus) and peripheral nervous system [37, 38]. Our findings suggest that nicotine treatment during the adolescence period increased basal metabolism (food intake) and the weight of the animals through nicotine mediated reward system that affected brain energy metabolism regulation [38]. Although nicotine is the major addictive substance in tobacco smoke, our results show that cadmium participates in the change in caloric intake that affect weight and energy expenditure (Figures 1 and 2). Our findings show that nicotine use increased the body weight and calorie intake in female adolescent mice; contrary to the idea that nicotine in tobacco is responsible for the weight loss seen in smokers [39, 40]. In addition, our results prove that a combination of nicotine and cadmium might be responsible for the weight loss associated with tobacco smoke as shown in Figures 1 and 2 (nicotine-cadmium treatment). Thus we deduce from these findings that changes observed in tobacco smoke are not exclusively nicotinic but a combination of cholinergic-signaling and other associated cellular mechanisms involving cadmium.

An increase in metabolism induced by nicotine is often associated with an increased motor function [41]. A model that addresses such function is linked with the ability of nicotine to stimulate nicotine receptors at the neuromuscular junctions and increase the release of epinephrine [42, 43]. As a result, glucose uptake increases in the muscles, thereby facilitating an increase in motor function [44]. Although this feature of nicotine use has been explored in Parkinson's disease (PD) therapeutics, it is often associated with increased anxiety due to the central effect of epinephrine [45]. Similarly, other studies have reported that smokers show a late onset of motor dysfunctions in PD when compared to nonsmokers due to the ability of nicotine to modify dopaminergic (D_1_) receptor dynamics following degeneration of dopaminergic neurons in the striatum [46–48]. However, this effect is not exclusive to the effect of nicotine on motor function. Recent findings suggest that nicotine modulates intrasynaptic dopaminergic release as part of its reward system. In addition, the D_1_ receptors observed in the striatum are also upregulated in the reward related brain regions [49].

Although nicotine increased motor function, subsequent analysis of animals treated with both nicotine-cadmium and cadmium showed a decline in motor functions when compared with the control and the nicotine group (Figure 4). The role of cadmium in inhibition of tissue respiration is suspected to be involved in the inhibition of synaptic function and transmission, thus blocking nicotinic synaptic effects in prolonged tobacco use (Figure 4). Similarly, Bierbower and Cooper demonstrated that carbon dioxide (CO_2_) produced via inhibition of tissue respiration blocks autonomic responses and inhibits glutamate neurotransmission at the neuromuscular junctions. Other studies have shown that cadmium is involved in the blockade of synaptic inputs at neuromuscular junctions and the central nervous system via upregulation of inhibitory neurotransmitter (GABA) and cellular carbon dioxide, thus accounting for its effect in reducing motor function when coadministered with nicotine or solely [50, 51].

Primary targets for the central effect of nicotine are receptors involved in the reward system and memory [52–54]. Similar to our findings, other experiments involving the post natal exposure of mice to nicotine between P28 and P43 have shown that gender variations exist in the observed memory functions and anxiety linked behaviors. While no significant change was observed in males, female animals showed an increased sensitivity to nicotine induced memory loss and anxiety [55]. Similarly, cadmium induced toxicity is capable of facilitating synaptic blockade and oxidative stress which mediates degenerative changes and memory loss. However, the effects of both nicotine and cadmium in adolescent have been linked with memory loss during use and after withdrawal [56]. Other documented evidence of tobacco-mediated memory loss includes depletion of nicotinic receptors in the prefrontal cortex [57, 58], increase in number of immature hippocampal neurons [59, 60], and inhibition of synaptic transmission both in the prefrontal cortex and hippocampus [59, 61].

### 5.1. Public Health Significance

Social or societal stress is often associated with adolescent humans who often engage in the abuse of nicotine, cannabinoids, amphetamine, and alcohol [62, 63]. Other reports have shown that tobacco use is most prevalent among adolescents and young adults between the ages of 13 and 25 years; furthermore, most start to smoke between the ages of 12 and 14 years [64, 65]. Although no significant difference was observed between the male and female adolescent smoking rates [66], females, however, show more susceptibility to nicotine-tobacco reward and addiction [31]. The long-term effect of tobacco use in adolescent females includes addiction [31, 66], sexual development [28], attention and memory deficits [67], and dependence during pregnancy [68].

## 6. Conclusion

We conclude that nicotine and cadmium when administered separately induced an increase in metabolism, food intake, and weight in adolescent female mice. However, the decrease in weight associated with tobacco smoke is as a result of nicotine-cadmium synergy rather than just nicotine. Also, nicotine increased motor function while cadmium and a combination of cadmium and nicotine decreased motor function due to synaptic inhibition by cadmium. Nicotine, cadmium, and nicotine-cadmium treatment decreased memory function, induced synaptic blockade, and possibly downregulated nicotinic receptors in the prefrontal cortex and hippocampus.
